# Novel HCN2 Mutation Contributes to Febrile Seizures by Shifting the Channel's Kinetics in a Temperature-Dependent Manner

**DOI:** 10.1371/journal.pone.0080376

**Published:** 2013-12-04

**Authors:** Yuki Nakamura, Xiuyu Shi, Tomohiro Numata, Yasuo Mori, Ryuji Inoue, Christoph Lossin, Tallie Z. Baram, Shinichi Hirose

**Affiliations:** 1 Department of Pediatrics, Faculty of Medicine, Fukuoka University, Fukuoka, Japan; 2 The Research Institute for the Molecular Pathomechanisms of Epilepsy, Fukuoka University, Fukuoka, Japan; 3 Department of Pediatrics, Chinese PLA General Hospital, Beijing, China; 4 Department of Synthetic Chemistry and Biological Chemistry, Graduate School of Engineering, Kyoto University, Kyoto, Japan; 5 Department of Physiology, Faculty of Medicine, Fukuoka University, Fukuoka, Japan; 6 Department of Neurology, School of Medicine University of California Davis, Sacramento, California, United States of America; 7 Departments of Anatomy & Neurobiology, Pediatrics, and Neurology, University of California Irvine, Irvine, California, United States of America; Sackler Medical School, Tel Aviv University, Israel

## Abstract

Hyperpolarization-activated cyclic nucleotide-gated (HCN) channel-mediated currents, known as *I*_h_, are involved in the control of rhythmic activity in neuronal circuits and in determining neuronal properties including the resting membrane potential. Recent studies have shown that HCN channels play a role in seizure susceptibility and in absence and limbic epilepsy including temporal lobe epilepsy following long febrile seizures (FS). This study focused on the potential contributions of abnormalities in the HCN2 isoform and their role in FS. A novel heterozygous missense mutation in *HCN2* exon 1 leading to p.S126L was identified in two unrelated patients with FS. The mutation was inherited from the mother who had suffered from FS in a pedigree. To determine the effect of this substitution we conducted whole-cell patch clamp electrophysiology. We found that mutant channels had elevated sensitivity to temperature. More specifically, they displayed faster kinetics at higher temperature. Kinetic shift by change of temperature sensitivity rather than the shift of voltage dependence led to increased availability of *I*_h_ in conditions promoting FS. Responses to cyclic AMP did not differ between wildtype and mutant channels. Thus, mutant HCN2 channels cause significant cAMP-independent enhanced availability of *I*_h_ during high temperatures, which may contribute to hyperthermia-induced neuronal hyperexcitability in some individuals with FS.

## Introduction

Hyperpolarization-activated cyclic nucleotide-gated (HCN) channels are nonselective cation channels in the heart and brain [1]–[3], and are encoded by four genes (*HCN1*, *2*, *3*, and *4*) [4]. HCN2 protein is expressed in regions including thalamus, brainstem, cerebral cortex, hippocampus, and amygdala [5], [6]. In hippocampus and cortex, HCN2 may co-assemble with HCN1, at least in part, to form heterotetrameric channels [7]–[13], which contributes to native *I*_h_, a hyperpolarization-activated cation current. Unlike in the thalamus, where *I*_h_ contributes to regulating rhythmic activity [1], [14], hippocampal and cortical *I*_h_ contributes to the resting membrane potential [1], [15], [16], and is involved in dendritic summation and other parameters of neuronal excitability [17]–[20].

A role for *I*_h_ in abnormal neuronal excitability has been documented [14], [17], [19], [21]–[25]. In addition to thalamocortical seizures [14], [26], HCN channels have been implicated in limbic seizures [25], [27]–[29], including those that arise after prolonged febrile seizures [27]. In support of a role of HCN2 channels in epileptic seizures, a mutation in *HCN2* was recently causally linked to general epilepsy [30].

Febrile seizures (FS) are the most common seizures in young children, and mutations in ion channels have been found in variants of FS that include epilepsy such as Dravet syndrome and Genetic epilepsy with FS plus (GEFS+), a clinical subset of FS [31]–[34]. However it remains unknown if abnormalities in HCN channel function contribute to typical FS.

The aims of this study were to (1) identify novel HCN2 mutations in children with FS, (2) examine differential temperature sensitivity between wildtype and mutant HCN2, and (3) examine the putative role of HCN2 mutations in FS. We analyzed *HCN2* for mutations in 160 children with FS, and identified one novel mutation in two individuals with FS. Characterization of the HCN2 mutant channels showed that the activation kinetics of mutant HCN2 were faster in hyperthermic environments, compared with the wildtype, increasing the availability of *I*_h_. Because HCN2-mediated *I*_h_ promotes neuronal firing, the results suggest that, in the presence of mutant HCN2, fever would induce neuronal hyperexcitability promoting FS.

## Materials and Methods

### Ethics statement

The present study was approved by the Ethics Review Committees of Fukuoka University. Parents of each patient and the parents themselves provided signed informed consent before the study.

### Sample collection and mutation screening

This study was performed in Japan. 160 Japanese children diagnosed with FS based on the guidelines of the American Academy of Pediatrics [35] and 125 healthy control subjects were examined. The eight exons and exon/intron boundaries of HCN2 were amplified by polymerase chain reaction (PCR). The sequences were analyzed using a direct sequencing method with a 3130*xl* Genetic Analyzer (Applied Biosystems).

### *HCN2* mutagenesis

Human HCN2 cDNA was a gift from Dr. A. Ludwig (Friedrich-Alexander-University, Erlangen-Nuremberg, Germany). This construct contains the *HCN2* open reading frame described by GenBank reference sequence NM_001194.3. Site-directed mutagenesis employed double-overlap PCR. The mutagenic primers were (forward) 5′-GAGCTCGGATCCACTAGTCCAGTGTGGTGG-3′ and (reverse) 5′-GCCCCGCGGCACaAGAACGACACCT-3′, where the altered base (c.377C>T) encoding the p.Ser126Leu (abbreviated as S126L hereafter) exchange is underlined and shown in lowercase letters.

### Expression of HCN channels and electrophysiological recordings

HEK293 cells were grown in Eagle's Minimum Essential Medium (MEM) containing 0.1 mM non-essential amino acids and 10% fetal bovine serum at 37°C in a humidified 5% CO_2_ environment. Transient expression of HCN2 was achieved by transfection of 1.0 µg plasmid DNA with Lipofectamine2000 (Invitrogen); pIRES2-EGFP (Clontech, Mountainview, CA) was co-transfected as a fluorescence marker in a 1∶10 mass ratio. In experiments where wildtype (W) and mutant DNA (M) were transfected simultaneously, equal amounts of both were transfected totaling 1.0 µg. Assuming random assembly and equal subunit supply, stochastically, this produced a mix of different channel types with the following distribution: MMMM and WWWW: 6.25% each; MWWW and WMMM in all 4 iterations 25% each, MMWW: 37.5% [36]. Three hours after transfection, the cells were dissociated with 0.25% trypsin/EDTA (Invitrogen) and seeded onto coverslips.

Electrophysiological analyses commenced 24–48 hrs after transfection on a fluorescence-enabled inverted microscope (Nikon, Japan) equipped with a temperature-controlled TC-324B recording chamber (Warner Instruments, Holliston, MA). The cells were continually superfused with extracellular solution containing (in mM): 110 NaCl, 30 KCl, 0.5 MgCl_2_, 1.8 CaCl_2_, and 5 HEPES, at pH 7.4 (NaOH). Recording pipettes were pulled from borosilicate glass on a Narishige PC-10 (Japan), and fire-polished on a Narishige MF-830 microforge. Filled with internal solution comprising (in mM) 130 KCl, 10 NaCl, 0.5 MgCl_2_, 1 EGTA, and 5 HEPES, at pH 7.4 (KOH), the pipettes had resistances of ∼3 MΩ. The liquid junction potential was negligible (∼3 mV, [37]) and corrected, along with the pipette capacitance, using the amplifier's circuitry (EPC-8, Heka Instruments, Bellmore, NY) prior to recording. Data sampled at 7 kHz were 7-pole Bessel filtered at 3 kHz and digitized with a PCI-6221 digitizer (National Instruments, Austin, TX). Series resistance was compensated by 40–70%. All experiments utilized a holding potential of −40 mV, which approximated the K^+^ Nernst potential (∼−38 mV) between 25 and 38°C. Cellular capacitance measurements relied on the amplifier's readout after cell capacitance compensation. Leak subtraction was not necessary because the holding current at *E_K_*_+_ (−40 mV) was commonly ≤10 pA.

### Fitting and statistical analysis

Data were analyzed off-line using the Strathclyde Electrophysiological Software (John Dempster, University of Strathclyde, Glasgow, UK, downloadable at http://spider.science.strath.ac.uk/sipbs/software_ses.htm). Peak amplitudes from tail currents acquired immediately after stepping back to the holding potential (−40 mV) were normalized to the maximal current, plotted against the test potential, and fitted with a Boltzmann function
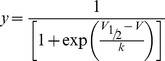
where *y* is the normalized tail current, *V* is the test potential, *V*_1/2_ is the half-activation potential and *k* is the slope factor. Activation kinetics were fitted with the double-exponential function y = *A_fast_*·exp(−*t*/*τ_fast_*)+*A_slow_*·exp(−*t*/*τ_slow_*)+*C*. Data are presented as mean ± SEM. Statistical analysis employed one-way ANOVA, and significance was set at *p*<0.05.

## Results

### *HCN2* gene analysis

**Figure 1:**
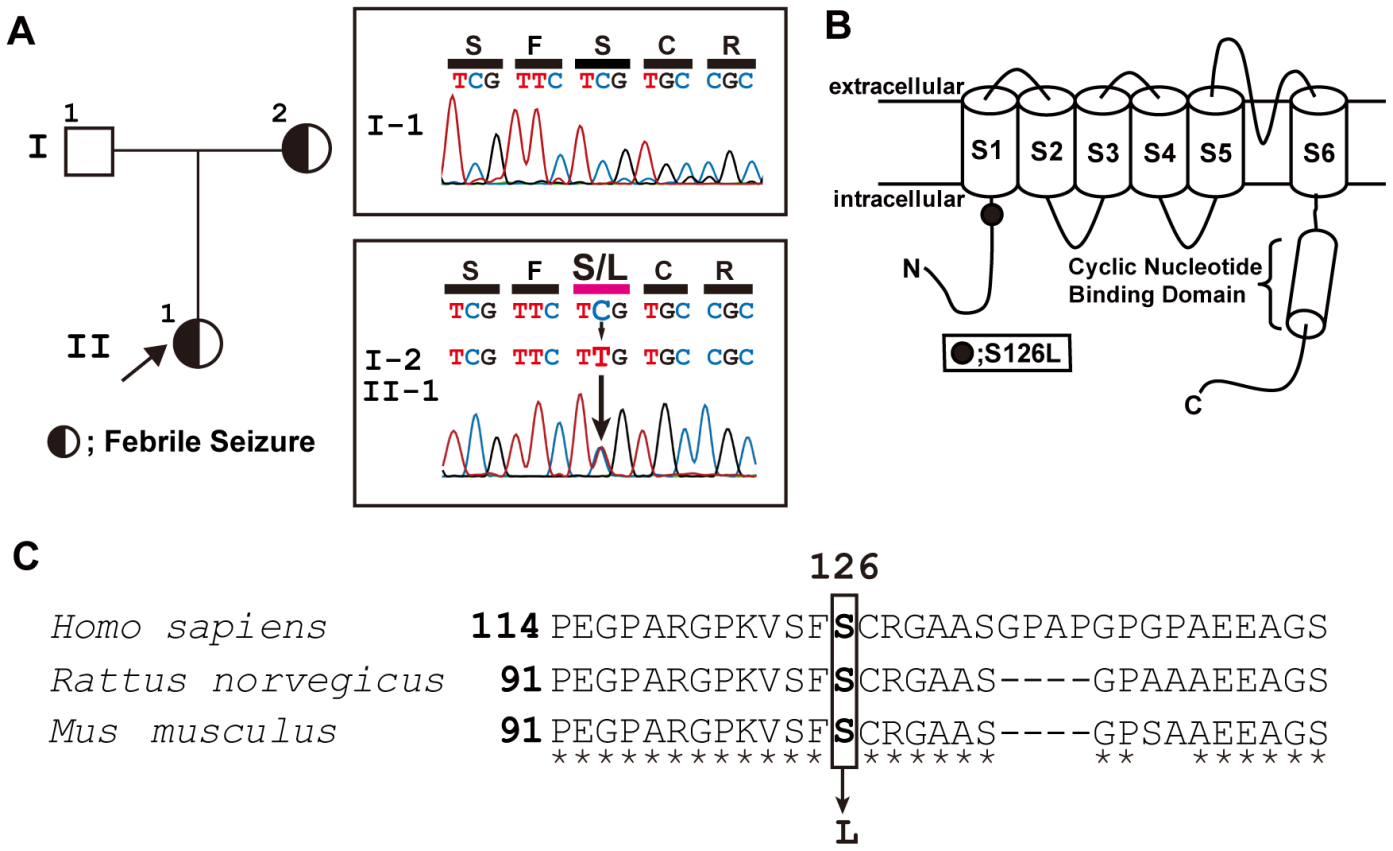
Genetic analysis of S126L. ***A left***, Family pedigree; the proband is indicated by an arrow. *A right* **,** Double-strand partial sequencing data for *HCN2* along with the amino acid translation for the unaffected father (I-1) and the mother and child with febrile seizure (I-2 and II-1). The missense mutation c.377C>T (S126L) is indicated by the arrow. ***B*** **,** Putative transmembrane topology of one HCN2 subunit showing the approximate position of S126 in the N-terminus. ***C*** **,** Sequence alignment of the N-terminus. The rectangle indicates the altered residue.

An *HCN2* mutation was identified as a heterozygous missense mutation (c. 377C>T) in two unrelated children with FS. The mother (I–2), who also suffered from FS in childhood, presented with the same *HCN2* alteration as her child (II–1); the father (I–1), unaffected, did not, suggesting that the change was inherited (Fig. 1 *A*). The mutation is predicted to substitute a polar serine at position 126 with a non-polar leucine (S126L). Position 126 is within the intracellular N–terminus, close to the S1 trans-membrane segment (Fig. 1*B*). The amino acid in this position varies among the four different human HCN channels. However, an alignment of HCN2 protein sequences across species shows that p.Ser126 is evolutionarily conserved (Fig. 1*C*). This is reiterated by the results of a SIFT *in silico* analysis, which predicts S126L to be damaging to HCN2 function [38]. Mutations in other FS candidate genes such as *SCN1A*, *SCN2A*, *SCN1B*, *SCN2B*, *GABRA1*, *GABRB2*, and *GABRG2* were not found in any of the patients.

### Electrophysiological characterization at 25°C and effects of temperature on wildtype, S126L and heteromeric HCN2 channels

**Figure 2:**
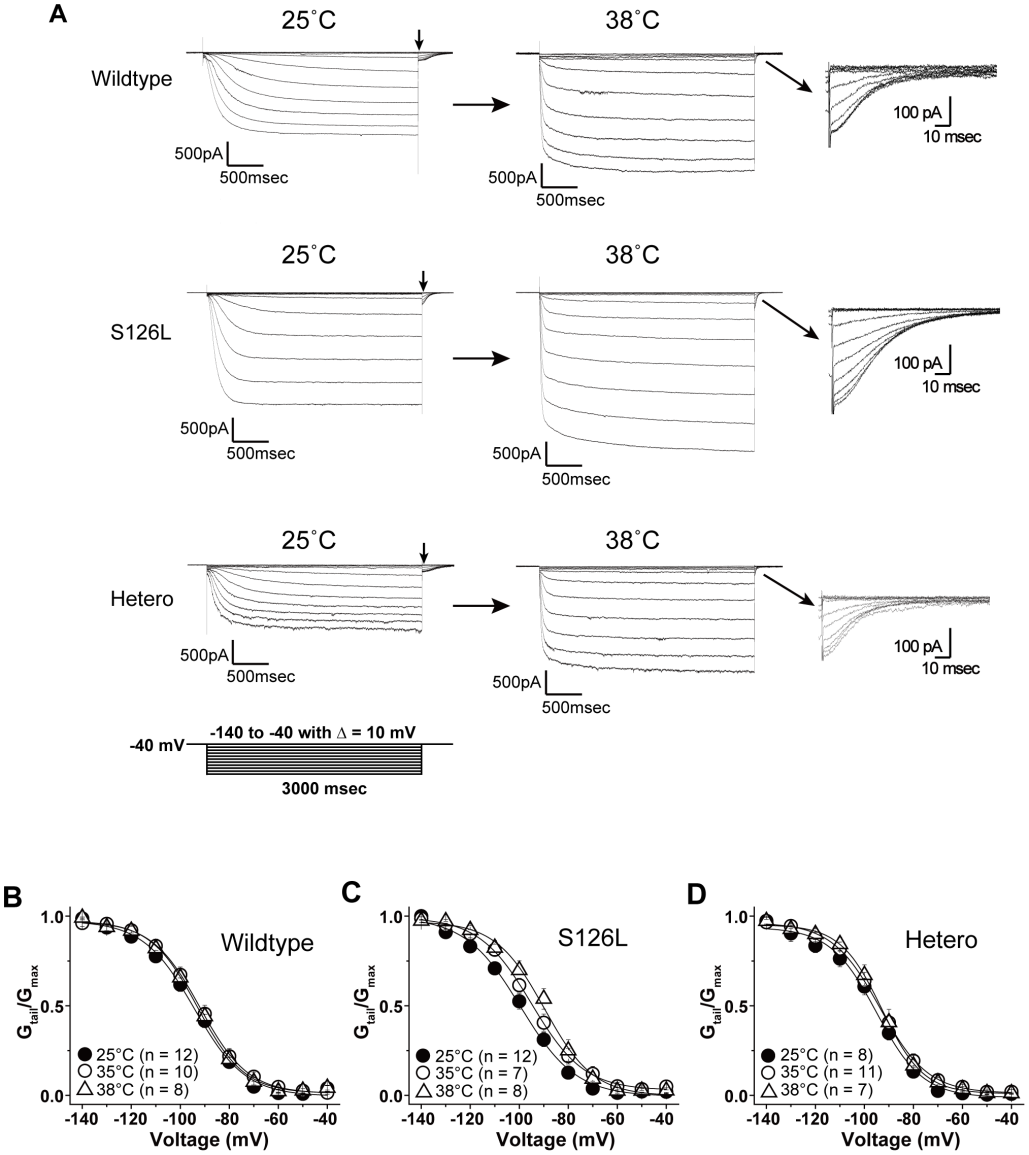
Current traces and activation curves of HCN2 wildtype, S126L and heretomeric channels at 25, 35 and 38°C. ***A***, Whole-cell currents of HCN2 wildtype (top), S126L (middle), and heteromeric (bottom) channels at 25 and 38°C. The currents were recorded in response to voltage pulses from a holding potential of −40 mV to test potentials between −140 to −40 mV. ***B, C, D*** **,** Conductance-voltage curves for wildtype, S126L and heteromeric channels at 25, 35 and 38°C. Continuous lines show Boltzmann fits of the experimental data.

FS, by definition, occur during fever. If S126L causes or contributes to the phenotype, one would expect that evidence of HCN2 dysfunction presents itself preferentially at or above 38°C. To examine the effect of fever on wildtype, wildtype/S126L (heteromeric) channels, and S126L homomeric channels, we recorded whole cell currents at 25 and 38°C, using 3-s voltage steps to hyperpolarizing potentials (−140 to −40 mV) from a −40 mV holding potential (Fig. 2 *A*). S126L and heteromeric channels exhibited a voltage-dependent inward current that resembled wildtype channels at 25°C and 38°C. Cells stimulated in this fashion produced voltage-dependent inward currents followed by tail currents when stepped back to the −40 mV hold. We used the tail currents (Fig. 2 *A*, arrows) to deduce the channels' voltage dependence and voltage sensitivity of activation (expressed in Table 1), by normalizing to the maximal amplitude and fitting it to a Boltzmann function (Fig. 2*B, C, and D*).

**Table 1:** *V_1/2_* for wildtype, S126L and heteromeric channels at 25°C and 38°C.

	25°C		38°C		Δ change
	*n*	*V_1/2_* (mV)	*n*	*V_1/2_* (mV)	
**wildtype**	12	−95.1±1.9	8	−92.7±2.0	2.4
**S126L**	12	−99.0±2.1	8	−90.1±2.6	8.9*
**hetero**	8	−94.9±1.3	7	−91.5±2.9	3.4

Data are mean ± SEM. Δ = (*V_1/2_* at 38°C)−(*V_1/2_* at 25°C).

* indicates *p*<0.05 compared with the wildtype.

The half maximal activation voltages (*V_1/2_*) at 25°C for wildtype (n = 12), S126L (n = 12) and heteromeric channels (n = 8) were −95.1±1.9, −99.0±2.1, and −94.9±1.3 mV, respectively, and the respective slope factors (*k*) were 10.3±0.9, 10.3±0.6, and 9.8±1.9. *V_1/2_* at 38°C for wildtype (n = 8), S126L (n = 8) and heteromeric channels (n = 7) were −92.7±2.0, −90.1±2.6 and −91.5±2.9 mV, respectively, and *k* were 9.9±1.5, 10.0±1.0 and 8.1±0.7.

**Figure 3:**
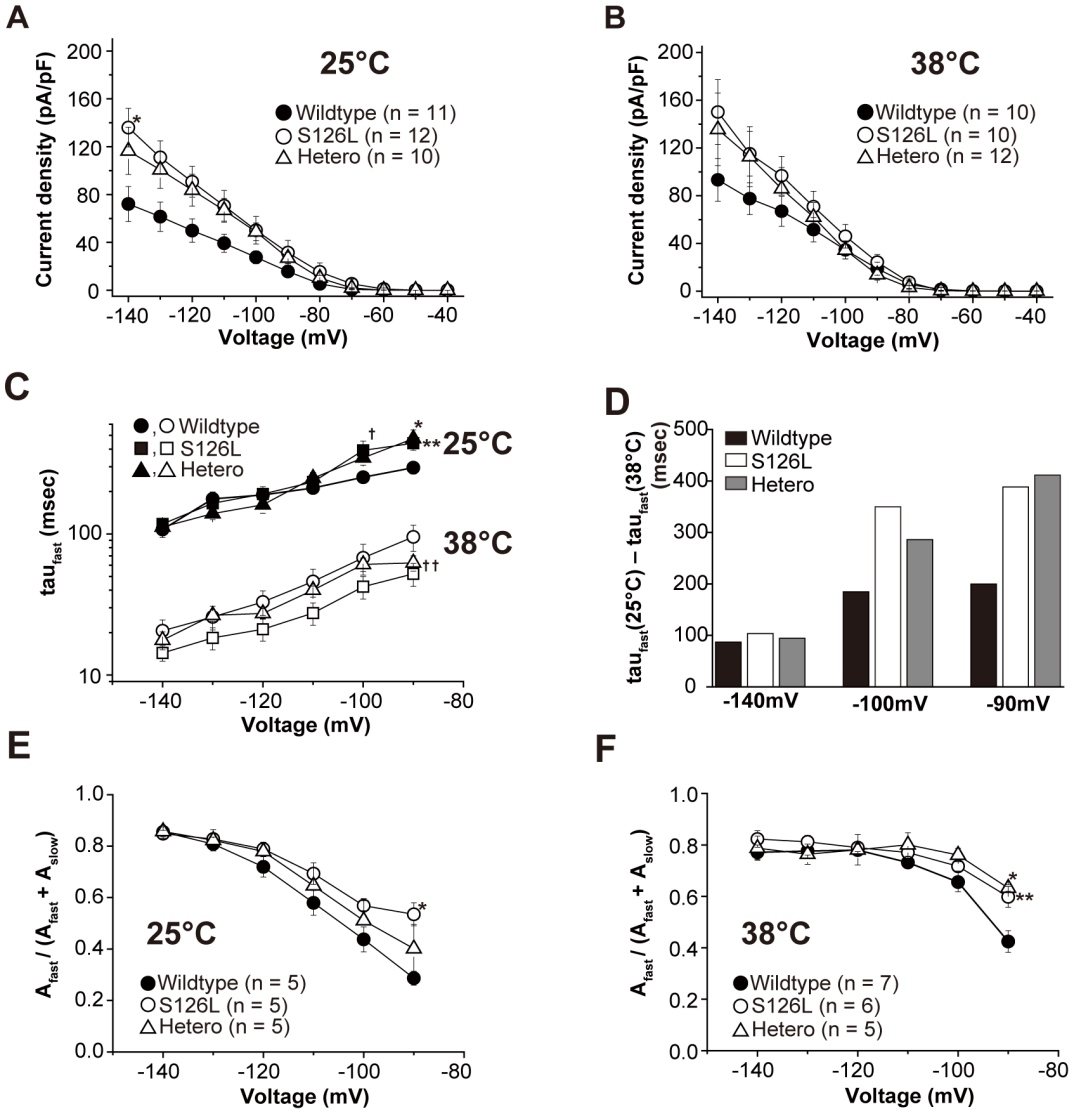
Kinetic properties in HCN2 wildtype, S126L and heteromeric channels at 25 and 38°C. ***A, B***, Current density at 25 (***A***) and 38°C (***B***). ***A*** **,** The current density of S126L channels at 25°C is significantly larger than that of wildtype at −140 mV (^*^ *p*<0.05). ***C*** **,** Fast activation time constants for wildtype, S126L and heteromeric channels at 25 and 38°C. ^†^ denotes tau fast of S126L channels at 25°C is significantly larger than that of wildtype channels at −100 mV. ^*^ denotes tau fast of S126L channels at 25°C is significantly larger than that of wildtype channels at −90 mV. ^**^ denotes tau fast of S126L channels at 25°C is significantly larger than that of heteromeric channels at −90 mV. ^††^ denotes tau fast of S126L channels at 38°C is significantly smaller than that of wildtype channels at −90 and −100 mV (^†, *, **, ††^ *p*<0.05). ***D*** **,** Alterations in tau fast by increasing temperature at −140, −100 and −90 mV. ***E, F*** **,** Relative amplitudes of fast exponential component as function of voltage at wildtype, S126L and heteromeric channels at 25 (***E***) and 38°C (***F***). ***E*** **,** Relative amplitude of S126L channels at 25°C was significantly larger than that of wildtype channels at −90 mV (^*^ *p*<0.05). ***F*** **,** Relative amplitude of S126L and heteromeric channels at 38°C was significantly larger than that of wildtype channels at −90 mV (^*, **^ *p*<0.05).

No significant differences were found when *V_1/2_* of the wildtype, S126L and heteromeric channels were compared at same temperature. However, raising the temperature (from 25°C to 38°C) led to depolarizing shift (Δ*V_1/2_*) in S126L channels (Δ*V_1/2_* = +8.9 mV) that was significantly larger than in wildtype channels (Δ*V_1/2_*: 2.4 mV, p<0.05), which speaks for increased temperature sensitivity in the mutant (Table 1). The current-voltage relationship was determined by the steady-state current in response to step pulse stimuli for 3 seconds (Fig. 3 *A, B*). At 25°C, the current density of S126L at −140 mV (135.9±16.2 pA/pF) was significant larger than that of wildtype (72.1±14.6 pA/pF, *p*<0.05). Although there was no significant difference between the channels at 38°C, the current density of S126L at −140 mV (150.2±27.1 pA/pF) and that of heteromeric channels (135.6±30.4 pA/pF) were larger than that of wildtype (93.3±18.0 pA/pF, *p*>0.05) (Fig. 3B). Current traces of these channels were fitted by two exponential functions. The fast and slow time constants (tau fast and tau slow, respectively) describing the activation kinetics at 25°C were voltage-dependent and smaller (faster) at more hyperpolarized voltages (Fig. 3 *C*, top). Between −90 and −100 mV, tau fast was significantly larger (slower) in S126L channels (at −100 mV: 252.6±13 ms, n = 10 wildtype, compared with 391.9±64.3 ms, n = 10 S126L, *p*<0.05, Table S1). In −90 mV, tau fast was significantly larger in heteromeric channels (295.1±29.0 ms, n = 10 wildtype, compared with 473.9±72.4 ms, n = 7 hetero, *p*<0.05)

Tau fast and slow at 38°C (Fig. 3 *C*, bottom) also exhibited voltage dependence and were faster (smaller) than those observed at 25°C. In contrast to the observation at 25°C, tau fast of S126L and heteromeric channels was faster than that of wild type from −90 to −140 mV. This phenomenon was most prominent at −90 mV. To better characterize this behavior, we subtracted tau fast at 38°C from that of 25°C in −140, −100 and −90 mV (Fig. 3 *D*). In −140 mV, “tau fast (25°C) – tau fast (38°C)” (Δtau) of wildtype was nearly equal to that of S126L and heteromeric channels. However at −100 and −90 mV, Δtau of S126L and heteromeric channels was larger than that of wildtype channels. These results indicate that the shift of the activation kinetics for S126L and heteromeric channels due to raising the temperature is larger in mutant channels, and that this is more evident in relatively depolarized (physiological) voltage ranges.

Because the relative contribution of tau fast and slow to the activation kinetics influence channel behavior [7], [39], we calculated the relative amplitude of the fast and slow exponential components of *I*_h_ (Fig. 3 *E, F*, Table S1). At 25°C, in S126L channels, the contribution of the fast component was significantly higher at −90 mV compared with wildtype channels (Fig. 3 *E*, *p*<0.05). These data indicate that the fast component (*A*_fast_) in S126L channels is increased at depolarized voltages. The contribution of the fast component to total *I*_h_ in each channel at 38°C was less voltage dependent than at 25°C (Fig. 3 *F vs.* 3 *E*). The contribution of the fast component of S126L and heteromeric channels was significantly increased compared with wildtype channels at −90 mV (Fig. 3 *F*, *p*<0.05). These results suggest that the fast component (*A*_fast_) in S126L and heteromeric channels is increased at more physiological potentials and at hyperthermic temperatures, and that, compared to wildtype, S126L and heteromeric channels exhibit altered temperature sensitivity. In summary, the shift of the activation kinetics by a raise in temperature was highly influenced rather than that of the conductance and the voltage dependence.

### Comparison of temperature dependence in wildtype, S126L and heteromeric channels

**Figure 4:**
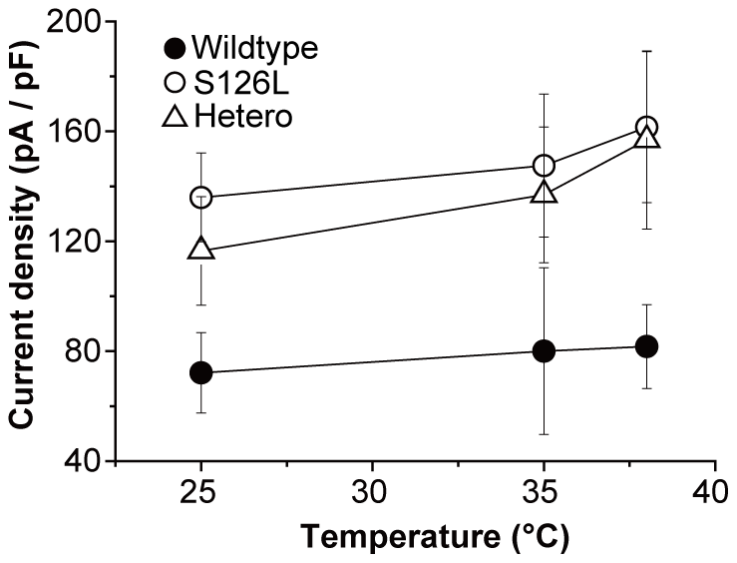
Temperature dependence of HCN2 wildtype, S126L and heteromeric channels. The effect of temperature on wildtype (n = 7–11), S126L (n = 7–12) and heteromeric (n = 7–10) channels at −140 mV. Elevated temperature raises the current densities in all channels, but only S126L-containing channels show a distinctly steeper slope from 35 to 38°C.

To evaluate the temperature dependence in each channel, we measured the *I*_h_ amplitude at 25, 35 and 38°C. Figure 4 shows that the current density was increased by a rise in temperature in all channels. Between 25 to 35°C, increased current density was similar in S126L (slope: 1.2±3.0 pA·pF^−1^·K^−1^), heteromeric (2.0±3.1 pA·pF^−1^·K^−1^) and wildtype (0.8±3.0 pA·pF^−1^·K^−1^) channels (Table S2). However, raising the temperature from 35 to 38°C - a temperature commonly associated with fever - led to highly divergent effects: whereas a gradual increase continued in the current density of wildtype (0.6±10.1 pA·pF^−1^·K^−1^) channel, a drastic, steep increase in current density occurred in the S126L (4.7±12.6 pA·pF^−1^·K^−1^) and heteromeric (6.7±14.7 pA·pF^−1^·K^−1^) channels. These results suggest that specific modulation by this mutation was occurred between 35 and 38°C.

### Role of cAMP in mutant HCN2 channel function

**Figure 5:**
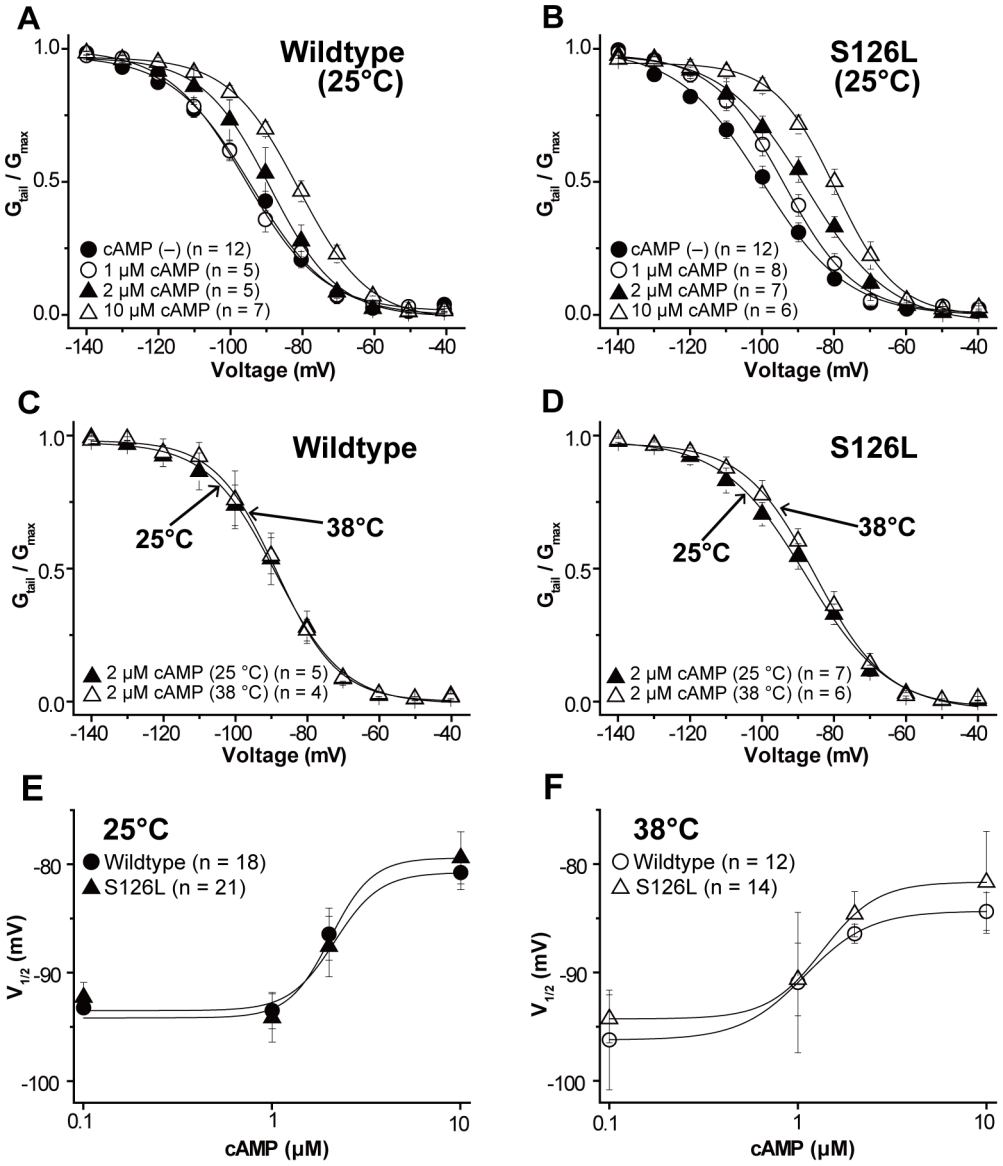
Effects of cAMP concentrations and raising temperature. ***A, B***, Conductance-voltage curves of wildtype (***A***) and S126L (***B***) channels in the absence and presence of cAMP at concentrations of 1, 2, and 10 µM. ***C, D*** **,** Comparison of conductance-voltage curves between at 25°C and at 38°C in 2 µM cAMP of wildtype (***C***) and S126L (***D***) channels. ***E, F*** **,** Hill fits of the cAMP dose-response as measured by half-maximal activation at 25°C and 38°C.

Because cAMP is a direct modulator of HCN channels [3], [40] and may influence the availability of *I*_h_ during hyperthermia, we investigated the effects of cAMP on S126L in comparison to wildtype channels. As expected, the activation curves of both channels shifted towards more depolarized potentials following application of cAMP at 25°C (Fig. 5 *A, B*). In addition, there was a tendency for higher sensitivity of the mutant channels to low doses (2 µM) of cAMP (Table S3, *p*<0.05).

More specifically, focused on 2 µM cAMP – the lowest dose leading to consistent changes in the activation curves in all channels at 38°C – resulted in a +3.5 mV(*p* = 0.40) right-shift of the activation curve for S126L channels. Wildtype channel, on the other hand, showed almost no shift (−0.1 mV, *p* = 0.98, Table S4). Although no significant difference was noted between the wildtype and S126L channels, this change produces clear conductance differences that enables the mutant to mediate more current (Fig. 5 *C*, *D*).

The data produced in Figure 5*A and B* were re-plotted to produce cAMP dose-response curves based on the half-maximal activation (Fig. 5*E, F*). Hill fits of the data show that S126L channels were activated at more positive voltages than wildtype channels at 38°C and that both channels at 38°C were activated by lower cAMP concentrations compared with 25°C. However *K_1/_*_2_ and *h* values for S126L channels were close to these values for wildtype channels (Table S5). These results suggest that the altered profile of S126L channels is a result of changes in voltage dependence and temperature sensitivity rather than in cAMP dependence.

## Discussion

Here we identified a novel heterozygous *HCN2* missense mutation (S126L) in children with FS and characterized its functional properties. We found increased current densities and accelerated kinetics in HCN2 channel with the S126L mutant. These alterations produce a mutant that allows more positive charge to enter the cell more quickly compared to healthy cells, specifically at elevated temperature, resulting in depolarization and excitation. Our results suggest that current mediated by S126L-containing channels may augment neuronal excitability during hyperthermia.

One may ask whether our findings are truly representative of what is seen in the patients when our data show the strongest effects in setups using only S126L channels, when the patients are heterozygous for the alteration. To emulate the patient internal environment, we used setups with a mix of wildtype and mutant DNA. This commonly produced behaviors that lay in between wildtype and purely-mutant, which may relate to the stochastic distribution of the ensuing channel possibilities. Closer analysis of possible subunit compositions for 1∶1 transfection experiments reveals that only 37.5% of the channels were composed of 2 wildtype and 2 mutant subunits. Exactly half of the channels (50%) contained 3 subunits of one type and the remaining subunit was of the other type. Most importantly, however, even in WT/mutant transfections, 12.5% of the channels were in fact homomeric, either wildtype or mutant. A “dose-dependent” effect is therefore to be expected, and our data confirm this, because all heteromeric channels produced electrophysiological deviations similar (usually somewhat reduced) or equal to what was seen in the setups using only mutant DNA. We have no way of examining the stoichiometry of the mutant channels in the native environment. It is uncertain, for example, whether, in the patients, S126L protein assembles with wildtype and/or mutant protein as readily as it does with healthy protein. Conversely, we cannot exclude that S126L does not enhance subunit interaction between the mutants, which could explain the augmented current densities as we elaborate below.

In most cortical and hippocampal neurons, *I*_h_ channels are constitutively open near the resting membrane potential [41]–[43], and contribute to its maintenance [1], [16]. In addition, *I*_h_ controls the membrane potential via a depolarizing inward current and by facilitating hyperpolarization [44], [45]. The large shift of the kinetics in homomeric S126L and heteromeric channels near the resting membrane potential at high temperatures could lead to early activation of *I*_h_ and disrupt this *I*_h_-based fine-tuning, promoting neuronal hyperexcitability.

In hippocampal CA1 neurons of immature rats, hyperthermia may reduce GABA_A_-receptor-mediated synaptic inhibition [46]. Such decrease, coupled with the shift in the activation kinetics of HCN2 S126L and heteromeric channels during hyperthermia could lead to an imbalance between neuronal excitation and inhibition, accelerating the development of FS.

Increased current densities at strongly hyperpolarized potentials have been reported before [24], [47], [48]. At this time, we cannot explain this phenomenon. Three factors contribute to macroscopic current (*I*), namely the number of contributing channels (*N*), single-channel current (*i*), and open probability (*p*). We deem it unlikely that either *i* or *p* are altered, simply based on the location of p.Ser126 within the HCN2 structure. We expect to find p.Ser126 in the periphery of the channel, although a definitive crystal structure is so far unavailable. This position would situate p.Ser126 far from the pore, which makes it hard to conceive an influence on the HCN2 single-channel conductance, which defines *i* in a voltage-dependent fashion. Similarly, p.Ser126 would be unlikely to impact the open probability of the channel. Unfortunately, enhanced surface expression is not a plausible explanation for the increase in *I*_h_ either, as this would imply a voltage-dependent change in the number of channels in the membrane. There is, however, the possibility of a change in functional-channel availability. How this could be mediated is currently unclear. Possible scenarios, given the residue's mapping to the N-terminus, include abnormal phosphorylation as well as alterations in protein-protein interactions. The latter is particular interesting, owing to a known importance of the N-terminus in inter-subunit interaction and formation of functional homo- and heteromeric channels [9]. The substitution of a polar serine with a nonpolar leucine might modify intermolecular forces or hydrogen bonds with other amino acids, leading to aberrant interaction with other HCN subunits required for tetramerization. Indeed, heteromerization of HCN2 with HCN1 [9], [10] is highly regulated in the brain [12], [13]. Augmented heteromerization after experimental FS [13] has been correlated with a shift in *I*_h_ properties that is similar to the one we found in S126L channels [24]. These data raise the possibility that S126L enhances HCN2∶HCN1 interaction, leading to aberrant channel function.

The mutation might interfere also with the interaction of HCN2 with auxiliary proteins. HCN channels interact with filamin [49], TRIP8b [50], [51] and enzymes such as p38 MAPK [52] and Src kinase [53]. Whereas the large majority of these proteins interact with HCN1 [54], tamalin, KCNE2, and others interact with HCN2. S126L might impair these interactions, enhancing a kinetic shift of the channel and promoting FS.

In conclusion, a mutation in HCN2 has been identified, with a potential to contribute to FS in a subgroup of children. It is now the 3^rd^ HCN mutation found in seizures and epilepsy, all of which affect HCN2. This underlies the importance of HCN channels to normal and aberrant neuronal excitability, and the specific properties of HCN2 that make mutations in these channels (or their absence) likely to result in a hyperexcitable network.

## Supporting Information

Table S1 **Kinetic parameters for HCN2 current activation at −90 mV based on double-exponential fits.** For the sake of clarity, only fast kinetic parameters are listed. * indicates *p*<0.05 versus wildtype.
(DOC)

Table S2 **Development of current density versus temperature.** The difference in the averaged current densities between two temperature points at −140 mV was calculated as a slope value.
(DOC)

Table S3 **cAMP sensitivity in wildtype and mutant channels: comparison of the shift in the voltage dependence of activation.** The Δ*V_1/2_* describes the cAMP-induced voltage shift in half-maximal activation compared to control levels. * indicates *p*<0.05 compared to the control.
(DOC)

Table S4 **Temperature dependence of cAMP sensitivity in wildtype and mutant channels.**
(DOC)

Table S5 **cAMP dose-response curves based on half-maximal activation voltages.**
(DOC)
