# Supplementary material for: Novel HCN2 Mutation Contributes to Febrile Seizures by Shifting the Channel's Kinetics in a Temperature-Dependent Manner
Source: PLoS One. 2013 Dec 4;8(12):e80376. doi: 10.1371/journal.pone.0080376 (PMC3851455; doi:10.1371/journal.pone.0080376)
Supplement: Table S1 — Kinetic parameters for HCN2 current activation at −90 mV based on double-exponential fits. For the sake of clarity, only fast kinetic parameters are listed. * indicates p<0.05 versus wildtype. (DOC) [file pone.0080376.s001.doc]

**Table S1. Kinetic parameters for HCN2 current activation at** −**90 mV based on double-exponential fits.**

|  | **25 ºC** | | |  | **38 ºC** | | |
| --- | --- | --- | --- | --- | --- | --- | --- |
|  | ***n*** | ***A_fast_ / ( A_fast_ + A_slow_)*** | ***tau_fast_* (msec)** |  | ***n*** | ***A_fast_ / ( A_fast_ + A_slow_)*** | ***tau_fast_* (msec)** |
| **wildtype** | 10 | 0.29 ± 0.02 | 295.1 ± 29.0 |  | 5 | 0.42 ± 0.04 | 95.4 ± 20.1 |
| **S126L** | 10 | 0.53 ± 0.04* | 440.4 ± 48.0 |  | 7 | 0.60 ± 0.04* | 52.2 ± 9.6* |
| **hetero** | 7 | 0.40 ± 0.09 | 473.9 ± 72.4* |  | 8 | 0.63 ± 0.01* | 62.4 ± 7.8 |

For the sake of clarity, only fast kinetic parameters are listed. * indicates *p*<0.05 versus wildtype.
