# Supplementary material for: Novel HCN2 Mutation Contributes to Febrile Seizures by Shifting the Channel's Kinetics in a Temperature-Dependent Manner
Source: PLoS One. 2013 Dec 4;8(12):e80376. doi: 10.1371/journal.pone.0080376 (PMC3851455; doi:10.1371/journal.pone.0080376)
Supplement: Table S2 — Development of current density versus temperature. The difference in the averaged current densities between two temperature points at −140 mV was calculated as a slope value. (DOC) [file pone.0080376.s002.doc]

**Table S2. Development of current density versus temperature.**

|  | **25 ⇨ 35 °C** | |  | **35 ⇨ 38 °C** | |
| --- | --- | --- | --- | --- | --- |
|  | ***n*** | **Slope (pA·pF^-1^·K^-1^)`** |  | ***n*** | **Slope (pA·pF^-1^·K^-1^)** |
| **wildtype** | 7 | 0.8 ± 3.0 |  | 7 | 0. 6 ± 10.1 |
| **S126L** | 10 | 1.2 ± 3.0 |  | 7 | 4.7 ± 12.6 |
| **hetero** | 7 | 2.0 ± 3.1 |  | 8 | 6.7 ± 14.7 |

The difference in the averaged current densities between two temperature points at −140 mV was calculated as a slope value.
