# Supplementary material for: Novel HCN2 Mutation Contributes to Febrile Seizures by Shifting the Channel's Kinetics in a Temperature-Dependent Manner
Source: PLoS One. 2013 Dec 4;8(12):e80376. doi: 10.1371/journal.pone.0080376 (PMC3851455; doi:10.1371/journal.pone.0080376)
Supplement: Table S3 — cAMP sensitivity in wildtype and mutant channels: comparison of the shift in the voltage dependence of activation. The ΔV1/2 describes the cAMP-induced voltage shift in half-maximal activation compared to control levels. * indicates p<0.05 compared to the control. (DOC) [file pone.0080376.s003.doc]

**Table S3.** cAMP sensitivity in wildtype and mutant channels: comparison of the shift in the voltage dependence of activation.

|  | **Wildtype** | | | **Δ*V_1/2_* (mV)** |  | **S126L** | | | **Δ*V_1/2_* (mV)** |
| --- | --- | --- | --- | --- | --- | --- | --- | --- | --- |
|  | ***n*** | ***V_1/2_* (mV)** | ***k*** |  |  | ***n*** | ***V_1/2_* (mV)** | ***k*** |  |
| **Control** | 12 | −95.1 ± 1.9 | 10.3 ± 0.9 | N.A. |  | 12 | −99.0 ± 2.1 | 10.3 ± 0.6 | N.A. |
| **1 μM cAMP** | 5 | −93.5 ± 1.7 | 10.0 ± 1.5 | +1.6 |  | 8 | −94.2 ± 2.2 | 9.8 ± 0.9 | +4.8 |
| **2 μM cAMP** | 5 | −90.1 ± 4.1 | 8.9 ± 1.1 | +5.0 |  | 7 | −89.4 ± 2.9 | 10.4 ± 0.6 | +9.6* |
| **10 μM cAMP** | 7 | −80.8 ± 1.6 | 9.7 ± 0.9 | +14.3* |  | 6 | −79.4 ± 2.4 | 8.2 ± 0.8 | +19.6* |

The Δ*V_1/2_* describes the cAMP-induced voltage shift in half-maximal activation compared to control levels. * indicates *p* < 0.05 compared to the control.
