# Supplementary material for: Novel HCN2 Mutation Contributes to Febrile Seizures by Shifting the Channel's Kinetics in a Temperature-Dependent Manner
Source: PLoS One. 2013 Dec 4;8(12):e80376. doi: 10.1371/journal.pone.0080376 (PMC3851455; doi:10.1371/journal.pone.0080376)
Supplement: Table S4 — Temperature dependence of cAMP sensitivity in wildtype and mutant channels. (DOC) [file pone.0080376.s004.doc]

**Table S4.** **Temperature dependence of cAMP sensitivity in wildtype and mutant channels.**

|  | **25 °C** | | |  | **38 °C** | | | **Δ*V_1/2_* (mV)** |
| --- | --- | --- | --- | --- | --- | --- | --- | --- |
|  | ***n*** | ***V_1/2_* (mV)** | ***k*** |  | ***n*** | ***V_1/2_* (mV)** | ***k*** |  |
| **wildtype** | 5 | −90.1 ± 4.1 | 8.9 ± 1.1 |  | 4 | −90.2 ± 3.8 | 8.7 ± 1.8 | −0.1 |
| **S126L** | 7 | −89.4 ± 2.9 | 10.4 ± 0.6 |  | 6 | −85.9 ± 2.8 | 10.1 ± 1.2 | +3.5 |
