# Supplementary material for: Novel HCN2 Mutation Contributes to Febrile Seizures by Shifting the Channel's Kinetics in a Temperature-Dependent Manner
Source: PLoS One. 2013 Dec 4;8(12):e80376. doi: 10.1371/journal.pone.0080376 (PMC3851455; doi:10.1371/journal.pone.0080376)
Supplement: Table S5 — cAMP dose-response curves based on half-maximal activation voltages. (DOC) [file pone.0080376.s005.doc]

**Table S5.** cAMP dose-response curves based on half-maximal activation voltages.

|  | **25 °C** | | |  | **38 °C** | | |
| --- | --- | --- | --- | --- | --- | --- | --- |
|  | ***n*** | ***K_1/2_* (μM)** | ***h*** |  | ***n*** | ***K_1/2_* (μM)** | ***h*** |
| **wildtype** | 18 | 2.1 | 3.6 |  | 12 | 1.1 | 2.5 |
| **S126L** | 21 | 2.0 | 3.9 |  | 14 | 1.4 | 3.0 |
